# Association of adipokines with blood pressure, arterial elasticity and cardiac markers in dialysis patients: cross-sectional analysis of baseline data from a cohort study

**DOI:** 10.1186/s12986-017-0185-3

**Published:** 2017-05-10

**Authors:** Wenjin Liu, Lei Jiang, Jianping Chen, Chaoqing Gao, Jianmei Zhou, Jiajun Zhou, Youwei Bai, Hong Chu, Wei Fan, Liang Wang, Zhuxing Sun, Xiurong Li, Junwei Yang

**Affiliations:** 1 Center for Kidney Disease, Second Affiliated Hospital of Nanjing Medical University, 262# North Zhongshan Road, Nanjing, 210003 China; 2 Department of Statistics Analysis, Affiliated Drum Tower Hospital, Nanjing University Medical School, Nanjing, China; 3 Department of Hemodialysis, Yijishan Hospital of Wannan Medical College, Wuhu, China; 4 Department of Nephrology, Luan People’s Hospital, Luan, China; 5 Department of Nephrology, Affiliated Yixing People’s Hospital, Jiangsu University, Yixing, China; 6 Department of Nephrology, Wuxi People’s Hospital, Nanjing Medical University, Wuxi, China; 7 Department of Blood Purification, The Third Affiliated Hospital of Soochow University, Changzhou, China

**Keywords:** Adipokines, Dialysis, Cardiovascular disease, Blood pressure, Arterial elasticity

## Abstract

**Background:**

Adipokines are a set of cytokines secreted by white adipose tissue that have been suggested to be involved in the development of cardiovascular diseases. We aimed to evaluate the cross-sectional associations of a panel of representative adipokines with cardiovascular measures in a cohort of hemodialysis patients.

**Methods:**

We measured plasma adiponectin, resistin, plasminogen activator inhibitor-1 (PAI-1), leptin, monocyte chemotactic protein 1 (MCP-1) and adipsin levels in 366 dialysis patients and 60 healthy controls. The associations of these adipokines with systolic blood pressure (assessed by ambulatory blood pressure monitoring), pulse wave velocity (PWV) and cardiac markers (BNP, NT-proBNP, Troponin I, Troponin T) in these patients were determined by general linear models with stepwise adjustment for covariates.

**Results:**

In unadjusted comparison with controls, dialysis patients showed increased adiponectin, resistin, MCP-1 and adipsin levels, decreased PAI-1 concentrations (all *p* <0.001) and similar leptin levels (*p* = 0.82). On adjustment for body mass index and diabetes, however, the PAI-1 level was comparable between group (*p* = 0.06), whereas leptin levels became significantly higher in the patients(*p* <0.001). Higher adiponectin, lower PAI-1 and leptin levels were associated with higher systolic blood pressure, even after extensive adjustment (all *p* ≤ 0.01). Adiponectin was also consistently and inversely associated with PWV in fully adjusted models (*p* = 0.003). Resistin, PAI-1, leptin and adipsin showed negative associations with one or more circulating cardiac markers (all *p* ≤ 0.02).

**Conclusions:**

We found significant associations between adipokines and cardiovascular measures. Our data suggest the possible involvement of adipokines in cardiovascular modulation in dialysis patients.

**Electronic supplementary material:**

The online version of this article (doi:10.1186/s12986-017-0185-3) contains supplementary material, which is available to authorized users.

## Background

Adipokines are a group of substances released from white adipose tissue and act as cytokines or hormones in vivo. With proteomic profiling approaches, to date there are over 600 secreted proteins classified as adipokines [1]. Previous studies have unveiled their roles in metabolic regulation and disorders [2]. Recent research interest in their roles in cardiovascular disease has established links between these substances and the cardiovascular system [3]. Several cardiovascular alterations, including hypertension, arterial stiffness and endothelial dysfunction, were associated with dysregulated circulating adipokine levels [4–6]. Interestingly, prior data suggested a “yin-yang” role of these bioactive proteins in the development of cardiovascular diseases [3].

Patients on hemodialysis constitute a disease population with strikingly high risk for cardiovascular morbidity and mortality [7]. Despite great efforts devoted to this area and progress of our knowledge in this area, mechanisms underlying this increasing risk remain to be fully understood [8, 9]. Exploring factors contributing to modulation of the cardiovascular system in dialysis patients is therefore important and may bring new insights. Though studies have been performed on certain adipokines in patients with kidney disease, little is known about their role in cardiovascular modulation in dialysis patients. Moreover, given that there exists a “reverse epidemiology” in dialysis patients, with higher BMI associated with improved survival [10], it is possible that adipokines may exert contrary effects on the cardiovascular system in these patients compared to the general population.

Given that prior studies evaluating the associations between adipokines and cardiovascular injury were restricted to non-kidney disease populations, we here explore their associations with cardiovascular measures in a group of dialysis patients. In this exploration analysis, we related a panel of adipokines to: (1) blood pressure (BP), as evaluated by interdialytic ambulatory blood pressure monitoring (ABPM); (2) arterial elasticity, as measured by carotid-femoral pulse wave velocity (PWV); and (3) circulating cardiac markers, including BNP, NT-proBNP, Troponin I and Troponin T. The aim of this study was to obtain cross-sectional evidence suggesting: (1) the contributions of adipokines to blood pressure regulation; (2) the effect of adipokines on vascular function and (3) the possible roles of adipokines in cardiac injury.

## Methods

### Study population

This is a cross-sectional analysis of baseline data from an ongoing prospective cohort study that aims to study vascular dysfunction in dialysis patients. The study included patients who met the following criteria: (1) aged 18-80 years old, (2) on maintenance hemodialysis (4 h/thrice weekly) over 3 months, and (3) who have reached their dry weight by clinical judgment. Exclusion criteria were as follows: (1) Malignant hypertension with systolic blood pressure (SBP) ≥ 180 mmHg or diastolic blood pressure (DBP) ≥ 110 mmHg; (2) Acute infection (respiratory, skin or any other type), acute heart failure or the acute phase of stroke (ischemic or hemorrhagic); (3) Decompensated liver cirrhosis or malignant tumor; (4) Amyloidosis or dilated cardiomyopathy; and (5) Internal jugular vein catheterization, previous fistula creation on the non-access arm or any other conditions that will affect the accuracy of peripheral arterial tonometry. Participants were recruited from six tertiary hospitals: The Second Affiliated Hospital of Nanjing Medical University (Nanjing, China), The Third Affiliated Hospital of Soochow University (Changzhou, China), Luan People’s Hospital (Luan, China), Affiliated Yixing People's Hospital of Jiangsu University (Yixing, China), Affiliated Wuxi People's Hospital of Nanjing Medical University (Wuxi, China), and Yijishan Hospital of Wannan Medical College (Wuhu, China); To compare adipokine levels between dialysis patients and normal subjects for the current study, we further recruited sixty apparently healthy subjects, frequency-matched to patients by age and sex, without any known history of cardiovascular or kidney disease as healthy controls. All participants provided written informed consent. The study was approved by the Institutional Ethical Committee of The Second Affiliated Hospital of Nanjing Medical University.

### Pulse wave velocity

Carotid-femoral PWV was measured on a midweek nondialysis day using the Complior Analyzer device (Artech Medical, France). Since the centers are in different cities and asking the patients to travel from city to city to get PWV measurement would be infeasible, all PWV measurement were performed by an experienced nurse from the Second Affiliated Hospital of Nanjing Medical University in each coordinating center. By this way the quality of PWV measurement was assured with inter-operator bias avoided. Patients were instructed to restrain from caffeine, meals and nitrates within 2 h before measurement (long-acting nitrates for 12 h). Patients rested for 10 min and then an experienced nurse from the Second Affiliated Hospital of Nanjing Medical University performed the test. Three probes were placed in a location with palpable pulse of the carotid, femoral and radial arteries. Ten consecutive recordings were averaged to calculate the transit time using the intersecting tangent algorithm as recommended [11]. Carotid-femoral distances were calculated as direct measurements multiplied by 0.8. Tolerance value was required to be <3 ms for a valid measurement.

### Blood pressure

BP was evaluated using interdialytic ABPM, the current gold standard for blood pressure determination in dialysis patients. Evaluation began after PWV measurement and ended before the next dialysis session. The SpaceLabs 90217 (SpaceLabs Medical Inc, Redmond, WA) monitor was used and programmed to measure BP every 20 min between 6:00 am and 10:00 pm and every 30 min between 10:00 pm and 6:00 am. Patients were instructed to follow their daily activities and to keep their arm still during measurements. Recordings were downloaded using the manufacturer’s software (SpaceLabs Report Manager System) and were further extracted and analyzed using SPSS 19.0 (SPSS Inc, Chicago, IL). For patients who had <6 ambulatory BP readings, predialysis blood pressures from dialysis records were collected and averaged over 2-weeks (6 times) before the ABPM and were used instead. For the purpose of exploring the association between adipokines and blood pressure, we used systolic blood pressure because it is the most potent blood pressure component for predicting adverse events in both the general population and in patients on hemodialysis [12, 13].

### Laboratory measurements

Blood samples were drawn through vascular access before dialysis treatment and sent to local laboratory departments at each coordinating centers immediately for routine clinical tests (including hemoglobin, albumin, total cholesterol, triglycerides, high-density lipoprotein cholesterol, low-density lipoprotein cholesterol, serum calcium, serum phosphorous and parathyroid hormone). Analyzers used for routine laboratory tests in each coordinating center were summarized in Additional file 1: Table S1. Another 4 ml blood sample was collected using an EDTA-K3 tube (Vacuette 454036; Greiner Bio-One GmbH, Austria) and centrifuged at 1500 g × 10 min to separate plasma within 30 min. For healthy controls, non-fasting blood samples were obtained for plasma separation after PWV measurement. All plasma was shipped to a core laboratory and stored at -80 °C until measurement.

Plasma adipokines (adiponectin, resistin, plasminogen activator inhibitor-1 (PAI-1), leptin, monocyte chemoattractant protein-1 (MCP-1) and adipsin), cardiac markers (BNP, NT proBNP, Troponin I, Troponin T) and C-reactive protein (CRP) were measured using the Milliplex Map assays (kit number: HCVD1MAG-67 k for BNP, NT proBNP, Troponin I; HCVD3MAG-67 k for adipsin, CRP; HCVD4MAG-67 k for Troponin T; HADK1MAG-61 k for adiponectin, PAI-1, resistin; HADK2MAG-61 k for leptin, MCP-1) (Merck Millipore, Shanghai, China) via the Luminex 200 system (Luminex Corporation, Texas, USA). Concentrations were calculated using the 5-parameter logistic curve fit. The results outside the range of the standards or the fit were further analyzed using a cubic spline. Intra- and inter-assay coefficients of variation were assessed using quality controls and presented in Additional file 1: Table S2).

### Statistical analyses

Kolmogorov–Smirnov test was used to check the normality of data distribution. Numerical variables were expressed as mean ± standard deviation for normally distributed parameters or median (interquartile range) for skewed distributions, while categorical variables were expressed as counts (%) or as ratio if n < 100. Numerical variables with skewed distributions were logarithm transformed for analysis as needed. Comparisons between two groups were performed using the Mann-Whiteny *U* test, Student’s *t* test or chi-square test as appropriate. For adjusted comparisons, analysis of covariance was used. Gender-pooled associations between adipokines and cardiovascular measures in the patients were determined by general linear models. We built three models for various adjustments for evaluating associations of adipokines with blood pressure and arterial elasticity. The first model served as a basic model where only age and sex were adjusted. The second model was further extensively adjusted for traditional and uremia-specific cardiovascular covariates. In Model-3, BMI was included as a covariate. CRP, as a reflection of inflammation, was entered into Model-3 for blood pressure and Model-2 for PWV since it was conventionally thought to be tightly associated with PWV, rather than blood pressure. For evaluation of associations between adipokines and the four cardiac markers, the same strategy was used to constructed the models, except for the basic model, which was omitted for conciseness. All statistical analyses were performed using SPSS 19.0 (IBM SPSS, Chicago, IL). Figures were generated using GraphPad Prism 6.0 (GraphPad Software Inc., San Diego, CA). A *p* value <0.05 was considered to be statistically significant.

## Results

Between July 2015 and July 2016, a total of 368 dialysis patients were recruited for the cohort. Plasma samples were available for 366 subjects. These patients constituted the study sample for the current analysis, except for analysis of associations between adipokines and PWV, from which five patients were further excluded due to invalid PWV measurements.

General characteristics of the study population and healthy controls are summarized in Table 1. The mean age of the study population was 52.5 years and 56.3% were male, both comparable to healthy controls (*p* = 0.29 for age and 0.85 for sex ratio, respectively). Body mass index (BMI) was significantly lower and diabetes mellitus was more prevalent in patients (*p* <0.01 for both). Dialysis patients were expected to have higher systolic blood pressure level (*p* <0.001).

**Table 1:** General characteristics of the study population and controls

	Study population *n* = 366	Controls *n* = 60	*p*
Age, yrs	52.5 ± 12.1	54.2 ± 9.1	0.29
Male	206 (56.3)	33/60^b^	0.85
BMI, kg/m^2^	21.9 ± 3.4	25.5 ± 2.9	<0.001
Current Smoker	80 (21.9)	11/60^b^	0.54
Dialysis Vintage, months	58.5 (31.0 – 89.0)	--	--
IDWG, kg	2.5 ± 0.9	--	--
Diabetes Mellitus	60 (16.4)	2/60^b^	0.005
History of CVD	38 (10.4%)	--	--
Use of antihypertensives	254 (69.4)	--	--
Use of ACEI/ARB	95 (26.0)	--	--
Statins	14 (3.8)	--	--
Weekly dose of EPO, IU	6000 (4000 - 9000)	--	--
Hemoglobin, g/L	108.7 ± 16.7	--	--
Albumin, g/L	39.5 ± 4.5	--	--
Total cholesterol, mmol/L	3.95 ± 0.94	--	--
Triglyceride, mmol/L	1.62 (1.17 – 2.28)	--	--
HDL cholesterol, mmol/L	1.00 ± 0.29	--	--
LDL cholesterol, mmol/L	2.06 ± 0.59	--	--
Phosphorus, mmol/L	1.81 ± 0.51	--	--
Calcium, mmol/L	2.28 ± 0.26	--	--
Parathyroid hormone, pg/mL	280.6 (109.7 – 559.5)	--	--
Systolic Blood Pressure	138.6 ± 22.7	124.1 ± 17.6	<0.001
Diastolic Blood Pressure	84.6 ± 12.7	83.0 ± 11.1	0.37
Heart Rate	77.0 ± 10.0	74.7 ± 13.9	0.12
PWV^a^	10.6 ± 3.5	--	--
CRP, ug/ml	5.40 (1.73 – 14.14)	--	--
BNP, pg/ml	280.7 (103.2 – 546.4)	--	--
NT proBNP, pg/ml	500.4 (281.2 – 787.0)	--	--
Troponin I, pg/ml	57.9 (20.6 – 158.3)	--	--
Troponin T, pg/ml	67.4 (22.8 – 117.5)	--	--

^a^ *n* = 361

^b^Presented as ratio since *n* < 100

Adipokine levels were presented and compared between dialysis patients and healthy controls in Additional file 1: Figure S1. In crude comparison (Additional file 1: Figure S1 A-F), dialysis patients showed increased adiponectin, resistin, MCP-1 and adipsin levels and decreased PAI-1 concentrations (*p* <0.001 for all). There was no difference regarding leptin levels (*p* = 0.82). To explore the confounding effect of BMI and diabetes status on adipokine levels, we compared log-transformed adipokine concentrations between patients and controls with adjustment for the two variables (Additional file 1: Figure S1 G-L). Adjustments for BMI and diabetes did not alter the relative level essentially for adiponectin, resistin, MCP-1 and adipsin. However, PAI-1 levels were comparable between the two groups with a marginal *p* of 0.06, whereas leptin levels became significantly higher in the patients than in healthy controls (*p* <0.001).

Table 2 presented the correlations of log-transformed adipokines with age, sex, BMI and diabetes status among the patients. PAI-1 and leptin were positively associated while adipsin was negatively associated with age (*p* ≤ 0.005 for all). Adiponectin, resistin and leptin were negatively associated while MCP-1 was positively associated with sex (coded as 0 = female and 1 = male) (*p* ≤ 0.03 for all). Higher BMI was associated with lower adiponectin concentrations (*p* <0.001) but higher PAI-1, leptin and MCP-1 levels (*p* <0.001 for all). There were inverse relationships between adiponectin and adipsin and diabetes (coded as 0 = No and 1 = Yes) (*p* ≤ 0.02 for both). It is worthy noting that most of the significant correlations were weak with correlation coefficient <0.3, except for correlations between BMI and adiponectin, PAI-1 and leptin (*r* = -0.54, 0.36 and 0.54 for adiponectin, PAI-1 and leptin, respectively).

**Table 2:** Correlations of log-transformed Adipokines with age, sex, BMI and diabetes status

	Age		Sex		BMI		Diabetes	
	*r*	*p*	*r*	*p*	*r*	*p*	*r*	*p*
Adiponectin	-0.07	0.17	**-0.25**	**<0.001**	**-0.54**	**<0.001**	**-0.12**	**0.02**
Resistin	-0.05	0.39	**-0.11**	**0.03**	0.03	0.62	-0.05	0.39
PAI-1	**0.28**	**<0.001**	0.01	0.92	**0.36**	**<0.001**	-0.02	0.67
Leptin	**0.20**	**<0.001**	**-0.29**	**<0.001**	**0.54**	**<0.001**	0.07	0.19
MCP-1	0.08	0.15	**0.14**	**0.008**	**0.19**	**<0.001**	-0.04	0.46
Adipsin	**-0.15**	**0.005**	0.06	0.23	0.08	0.13	**-0.15**	**0.003**

Sex was coded as 0 = female and 1 = male; diabetes was coded as 0 = No and 1 = Yes

Boldface entries are significant (p<0.05)

Table 3 demonstrated the association between log-transformed adipokines and systolic blood pressure and PWV. In a basic model adjusted for age and sex, all adipokines except for MCP-1 were significantly associated with systolic blood pressure (positively for adiponectin and negatively for resistin, PAI-1, leptin and adipsin, *p* ≤ 0.04 for all). In the more extensively adjusted model 2, the associations of adiponectin, PAI-1 and leptin remained essentially unchanged. When BMI and log-CRP were further included in model 3 as covariates, only associations between adiponectin and systolic blood pressure became non-significant. In the basic model (model 1), only leptin was negatively related to PWV. However, after further adjustment, adiponectin emerged as the sole adipokine associated, inversely, with PWV (*p* = 0.02 in model 2 and 0.003 in model 3, respectively).

**Table 3:** Associations of log-transformed Adipokines with systolic blood pressure and pulse wave velocity

	Model-1		Model-2		Model-3	
	ß	*p*	ß	*p*	ß	*p*
Systolic Blood Pressure						
Adiponectin	**7.80**	**0.001**	**6.02**	**0.01**	4.17	0.11
Resistin	**-10.16**	**0.04**	-5.07	0.25	-4.25	0.34
PAI-1	**-21.71**	**<0.001**	**-12.69**	**0.001**	**-10.83**	**0.008**
Leptin	**-8.89**	**<0.001**	**-6.89**	**<0.001**	**-6.14**	**0.005**
MCP-1	-5.89	0.30	-2.91	0.57	-1.38	0.79
Adipsin	**-18.20**	**0.009**	-5.16	0.44	-3.62	0.59
Pulse Wave Velocity						
Adiponectin	-0.34	0.30	**-0.80**	**0.02**	**-1.05**	**0.003**
Resistin	-0.84	0.23	-0.23	0.70	-0.22	0.72
PAI-1	-0.62	0.28	0.72	0.18	0.90	0.11
Leptin	**-0.58**	**0.02**	-0.39	0.12	-0.34	0.27
MCP-1	-0.78	0.33	-0.26	0.71	-0.18	0.80
Adipsin	-1.37	0.17	0.56	0.53	0.64	0.48

For associations between adipokines and systolic blood pressure: Model-1: adjusted for age and sex; Model-2: adjusted for covariates in Model-1 + dialysis vintage, current smoker, interdialytic weight gain, diabetes mellitus, history of cardiovascular disease, use of antihypertensives, log – weekly EPO dose, total and HDL cholesterol; Model-3: adjusted for covariates in Model-2 + BMI and log-CRP

For associations between adipokines and pulse wave velocity: Model-1: adjusted for age and sex; Model-2: adjusted for covariates in Model-1 + dialysis vintage, current smoker, interdialytic weight gain, diabetes mellitus, history of cardiovascular disease, use of antihypertensives, use of statins, total and HDL cholesterol, systolic blood pressure and log-CRP; Model-3: adjusted for covariates in Model-2 + BMI

Boldface entries are significant (p<0.05)

Associations of log-transformed adipokines with log-transformed circulating cardiac markers were also determined, as presented in Table 4. Resistin and PAI-1 were consistently and inversely correlated with BNP and NT-proBNP after extensive adjustment, whether BMI included as a covariate or not (*p* ≤ 0.02 for all). Negative associations were also found between leptin and NT-proBNP as well as adipsin and BNP (*p* ≤ 0.004 for all). For troponins, we found that PAI-1 was inversely associated with Troponin I while adipsin was negatively associated with both Troponin I and Troponin T (*p* ≤ 0.005 for all).

**Table 4:** Associations of log-transformed Adipokines with log-transformed Cardiac Markers

	Model-1		Model-2		Model-1		Model-2	
	ß	*p*	ß	*p*	ß	*p*	ß	*p*
	BNP				NT proBNP			
Adiponectin	0.08	0.24	0.04	0.57	0.03	0.48	0.03	0.59
Resistin	**-0.37**	**0.001**	**-0.36**	**0.002**	**-0.18**	**0.02**	**-0.18**	**0.02**
PAI-1	**-0.27**	**0.009**	**-0.24**	**0.02**	**-0.23**	**0.001**	**-0.23**	**0.001**
Leptin	-0.05	0.31	-6.84 × 10^-5^	0.99	**-0.10**	**0.002**	**-0.13**	**0.001**
MCP-1	-0.13	0.34	-0.10	0.45	-0.09	0.33	-0.09	0.35
Adipsin	**-0.52**	**0.003**	**-0.50**	**0.004**	-0.11	0.34	-0.11	0.36
	Troponin I				Troponin T			
Adiponectin	-0.11	0.10	-0.14	0.06	-0.05	0.54	-0.06	0.46
Resistin	-0.05	0.68	-0.05	0.69	0.04	0.76	0.04	0.75
PAI-1	**-0.32**	**0.005**	**-0.33**	**0.005**	-0.09	0.47	-0.09	0.50
Leptin	0.01	0.82	0.03	0.63	0.08	0.17	0.13	0.07
MCP-1	-0.13	0.35	-0.13	0.37	-0.22	0.18	-0.21	0.18
Adipsin	**-0.83**	**<0.001**	**-0.83**	**<0.001**	**-0.75**	**<0.001**	**-0.75**	**<0.001**

Model-1: adjusted for age, sex, dialysis vintage, current smoker, interdialytic weight gain, diabetes mellitus, history of cardiovascular disease, use of antihypertensives, use of statins, total and HDL cholesterol, systolic blood pressure and log-CRP

Model-2: adjusted for covariates in Model-1 + BMI

Boldface entries are significant (p<0.05)

## Discussion

In the current study, we explored the cross-sectional associations of adipokines with blood pressure, arterial elasticity and circulating cardiac markers in a cohort of patients on maintenance hemodialysis. To the best of our knowledge, this is the first report comprehensively relating a panel of representative adipokines to cardiovascular measures in this disease population.

Our results demonstrated the following: (1) Compared with healthy controls, circulating adiponectin, resistin, MCP-1 and adipsin concentrations were markedly elevated in dialysis patients while leptin levels remained comparable and PAI-1 levels were even reduced. Adjustment for BMI and diabetes could change the relative levels of the latter two adipokines significantly between groups. (2) Higher adiponectin, lower PAI-1 and leptin levels were associated with higher systolic blood pressures, even after extensive adjustment. (3) Adiponectin was consistently and inversely associated with PWV in fully adjusted models. (4) Resistin, PAI-1, leptin and adipsin were negatively associated with one or more circulating cardiac markers. These findings clearly suggest the participation of adipokines in modulation of the cardiovascular system of dialysis patients and need to be interpreted together with prior data from other study populations.

The six adipokines studied here have been implicated in various roles in different physiological and pathophysiological processes [2, 14]. The molecular weights range from 12.5-kDa (resistin) to 50-kDa (PAI-1). Due to reduced renal clearance, it was expected to see accumulated adiponectin, resistin, MCP-1 and adipsin in this cohort of dialysis patients, while decreased PAI-1 and similar leptin levels compared with the healthy controls were not expected. Both PAI-1 and leptin were positively associated with BMI and adjustment for BMI resulted in similar PAI-1 and increased leptin levels in patients compared to controls. This result indicated that the levels of the two adipokines were more susceptible to the influence of fat tissue mass rather than reduced renal clearance in dialysis patients, in whom malnutrition is prevalent and who are usually leaner than the general population [15]. This belief concerning leptin is supported by a previous work by Johansen et al. who found that plasma leptin levels were comparable among hemodialysis patients and healthy controls, and that they became significantly higher in patients after adjustment for percentage of body fat [16]. Data regarding PAI-1 levels in dialysis patients, however, were conflicting. Irish reported that dialysis patients had lower PAI-1 activity than control subjects despite comparable BMI while Segarra et al. concluded that both PAI-1 antigen levels and PAI-activity were elevated in dialysis patients [17, 18]. The explanation for this discrepancy is obscure and may involve sample distinction (plasma vs. serum), heterogeneity of patients or lack of a standardized protocol for measurement, thus warranting further exploration.

Adiponectin was the only adipokine associated with both blood pressure (positively) and PWV (negatively) in extensively adjusted models in our study. The bidirectional association of adiponectin with blood pressure and PWV is interesting. Previous studies usually suggested adiponectin as a protective adipokine for the cardiovascular system, with effects of lowering blood pressure and preserving arterial elasticity [6, 19–21]. There were two possible explanations for this finding: (1) Among dialysis patients, in whom hypertension is highly prevalent and usually poorly controlled [22], there may exist a feedback response between adiponectin and blood pressure. Elevation of blood pressure in these patients would induce increased secretion of adiponectin, which would, in turn, try to lower blood pressure and protect the arterial wall from stiffening; (2) Higher adiponectin levels may simply be detrimental to blood pressure regulation, as was observed in another case-control study of the general Chinese population [23].

Leptin is the first identified adipokine and is usually considered to elevate blood pressure. It can increase the activity of the sympathetic nervous system and activate the renin–angiotensin–aldosterone system, which will together lead to increased blood pressure [24, 25]. Previous studies demonstrated that leptin was associated positively with age, BMI and female gender in dialysis patients, which is corroborated by our findings. However, little is known about its association with blood pressure in these patients. Here we found that higher leptin levels were associated with lower blood pressure in dialysis patients, thus contradicting results from other study populations [5, 26]. Leptin levels were also inversely associated with NT-proBNP according to our results. These data indicate that higher leptin levels may confer lower cardiovascular risk. Indeed, this was confirmed by a small study of Chinese dialysis patients, which showed that low serum leptin predicts the risk of all-cause mortality [27].

PAI-1 is a serine protease inhibitor protein that functions as the principal inhibitor of tissue plasminogen activator. We showed that in dialysis patients, its circulating levels were comparable to those in healthy subjects and were negatively associated with blood pressure and several cardiac markers (BNP, Troponin I and Troponin T). Although a previous work demonstrated that increased plasma PAI-1 levels predicted cardiovascular risk in peritoneal dialysis patients [28], it needs to be noted that patients on peritoneal dialysis had significantly higher PAI-1 levels than controls or patients on hemodialysis [29].

In addition to the adipokines discussed above, we also noticed that there were consistent and negative associations between resistin and adipsin and circulating cardiac markers. Resistin is mainly expressed in macrophages from adipose tissue and is considered to contribute to insulin resistance and inflammation, while adipsin, also known as Factor D, is a key component of the alternative complement pathway. Data regarding roles of the two adipokines in the cardiovascular system of dialysis patients is scarce. Spoto et al. reported that resistin predicted cardiovascular events in a cohort of hemodialysis patients, but this effect was dependent on adiponectin levels [30]. The negative associations with cardiac markers in our study suggest possible beneficial effects of the adipokines but warrant further investigation.

Substantial work has been performed to investigate the physiologic and pathophysiologic mechanisms underlying the effect of different adipokines on the cardiovascular system and had been well summarized [2, 3, 31]. We want to note that most of the previous work was performed with adipokine levels in the physiologic range. Given the overt accumulation of some adipokines in dialysis patients as shown by our results, they may exert effects that differ from those in the general population. This was supported by a recent work by Zachariah et al. in which the authors examined the associations of several adipokines with vascular function in the community-based Framingham cohort [32]. They found that leptin, rather than adiponectin, was associated negatively with PWV. Moreover, the cross-sectional associations found in our study suggested the involvement of adipokines in cardiovascular modulation in dialysis patients, but can not confirm any specific roles of these substances in the cardiovascular system.

There are certain limitations of our study. First, as mentioned above, the cross-sectional design precludes any inference of causality. The associations of adipokines with cardiovascular measures should be interpreted with this caution and will need support from future evidence. Second, the study was limited to the Chinese population. Whether these associations persist in other races is uncertain, especially given the ethnic difference of adipokines demonstrated in previous studies [33, 34]. Third, routine biochemistry tests were not performed in healthy controls and whether these subjects have subclinical renal insufficiency was unknown. Finally, we used only BMI as a surrogate measure of fat depot, rather than any more direct measures.

## Conclusions

In conclusion, in a cohort of patients on maintenance hemodialysis we studied a panel of adipokines and their associations with several cardiovascular measures, including blood pressure, PWV and circulating cardiac markers. We found significant associations between adipokines and these measures. Our data suggest the possible involvement of adipokines in cardiovascular modulation in dialysis patients.

## Additional file

Additional file 1: Association of adipokines with blood pressure, arterial elasticity and cardiac markers in dialysis patients. (DOCX 3 kb)

## Abbreviations

ABPM: Ambulatory blood pressure monitoring
BMI: Body mass index
BP: Blood pressure
CRP: C-reactive protein
DBP: Diastolic blood pressure
MCP-1: Monocyte chemoattractant protein-1
PAI-1: Plasminogen activator inhibitor-1
PWV: Pulse wave velocity
SBP: Systolic blood pressure
