# Supplementary material for: Association of adipokines with blood pressure, arterial elasticity and cardiac markers in dialysis patients: cross-sectional analysis of baseline data from a cohort study
Source: Nutr Metab (Lond). 2017 May 10;14:34. doi: 10.1186/s12986-017-0185-3 (PMC5424399; doi:10.1186/s12986-017-0185-3)
Supplement: Additional file 1: — Association of adipokines with blood pressure, arterial elasticity and cardiac markers in dialysis patients. (DOCX 3 kb) [file 12986_2017_185_MOESM1_ESM.docx]

**Table S1. Analyzers used for routine laboratory tests in each coordinating center**

| Coordinating center | Analyzers | |
| --- | --- | --- |
|  | Blood cell analyzer | General chemistry |
| Second Affiliated Hospital of Nanjing Medical University | XS-800i (Sysmex Corporation, Kobe, Japan) | Cobas 8000 （Roche Ltd., Germany） |
| Yijishan Hospital of Wannan Medical College | BC-6900 (Mindray Bio-Medical Electronics Co, Shenzen, China) | Hitachi 7600 (Hitachi Ltd., Tokyo, Japan) |
| Luan People’s Hospital | XE-2100 (Sysmex Corporation, Kobe, Japan) | AU5821 (Beckman Coulter Inc., Brea CA, USA) |
| Affiliated Yixing People's Hospital, Jiangsu University | XN-10 (Sysmex Corporation, Kobe, Japan) | ADVIA 2400 (Siemens Healthcare, Germany) |
| Wuxi People's Hospital | XN-10 (Sysmex Corporation, Kobe, Japan) | DXC-800 (Beckman Coulter Inc., Brea CA, USA) |
| The Third Affiliated Hospital of Soochow University | XN-9000 (Sysmex Corporation, Kobe, Japan) | AU5800 (Beckman Coulter Inc., Brea CA, USA) |

**Table S2. Inter- and intra-assay coefficients of variation for biomarker measurement**

|  | Intra-assay CV* | | Inter-assay CV | |
| --- | --- | --- | --- | --- |
|  | QC1 | QC2 | QC1 | QC2 |
| adiponectin | 2.3% | 1.9% | 8% | 6% |
| resistin | 2.9% | 2.3% | 9% | 2% |
| PAI-1 | 4.2% | 4.4% | 4% | 17% |
| leptin | 1.2% | 2.2% | 25% | 12% |
| MCP-1 | 2.5% | 2.6% | 11% | 9% |
| adipsin | 2.6% | 1.0% | 13% | 9% |
| CRP | 4.1% | 2.5% | 28% | 15% |
| BNP | 2.6% | 6.3% | 19% | 8% |
| NT proBNP | 1.7% | 4.2% | 17% | 7% |
| TnI | 2.5% | 4.8% | 34% | 16% |
| TnT | 4.1% | 2.6% | 19% | 14% |

Abbreviations: CV, coefficients of variation; QC, quality control; PAI-1, plasminogen activator inhibitor-1; MCP-1, monocyte chemoattractant protein-1; CRP, C-reactive protein; BNP, B-type natriuretic peptide; NT proBNP, N-terminal pro-brain natriuretic peptide; TnI, Troponin I; TnT, Troponin T.

* Averaged over all plates

**Figure S1. Adipokine Levels in Patients and Healthy Controls**





A-F: Unadjusted comparison of adipokine levels between dialysis patients and controls; G-L: Comparison of log-transformed adipokine levels between dialysis patients and controls, adjusted for body mass index and diabetes.
